# How physical exercise influences academic burnout among Chinese “Double Non” college students: the chain mediation role of mobile phone addiction and learning engagement

**DOI:** 10.3389/fpsyg.2023.1289499

**Published:** 2024-01-05

**Authors:** Chao Jin, Chunhong Fan, Jinpeng Niu

**Affiliations:** ^1^College of Education, Qufu Normal University, Qufu, Shandong, China; ^2^Office of Academic Research, Jining University, Qufu, Shandong, China; ^3^Shandong Polytechnic College, Jining, Shandong, China

**Keywords:** physical exercise, mobile phone addiction, learning engagement, academic burnout, “Double Non” college students

## Abstract

**Background:**

With mental anxiety caused by the COVID-19 pandemic, the trend of “lying down” has become increasingly prevalent among Chinese college students such as not thinking ahead, decadent abandonment, and being weak daily. Particularly, Chinese “Double Non” college students are more likely to face academic burnout (AB) due to lower school satisfaction and after-degree employment ratio, in comparison to “Double First-Class” college students.

**Objectives:**

In view of this, the present study examined the structural relationships of physical exercise (PE), mobile phone addiction (MPA), learning engagement (LE), and AB among Chinese “Double Non” college students, aiming at exploring corresponding mechanism to provide supportive guidance for alleviating potential AB.

**Methods:**

This study adopted a cross-sectional survey approach among the sample of “Double Non” college students in China. We recruited 930 participants (272 men and 658 women) in the second half of the 2022–2023 academic year, all of whom completed questionnaires involving Physical Exercise Rating Scale, Mobile Phone Dependence Index Scale, Utrecht Work Engagement Scale-Student, and Maslach Burnout Inventory-Student Survey. A series of statistical analyses, including descriptive statistics, bivariate correlations, and direct and indirect effects among study variables, were proceeded based on the collected data.

**Results:**

The results showed that PE can not only directly and negatively influence AB, but also indirectly and negatively influence AB through the mediation of MPA as well as the chain mediation of both MPA and LE. While PE had no significantly indirect effect on AB via LE.

**Discussion:**

Physical exercise was proved to be an effective way to reduce MPA and then enhance LE, consequently leading to decreased AB in Chinese “Double Non” college students. The findings were discussed in light of related research, and implications and future directions were put forward for application in potential theoretical research and educational practice.

## Introduction

In recent years, studies related to academic burnout (AB) have attracted more and more attention among scholars (e.g., Wang, 2008; Gerber et al., 2013; Cazan, 2015; Li et al., 2021). AB mainly refers to a negative psychological syndrome caused by excessive academic pressure, embodying reduced enthusiasm toward learning (Wang, 2008; Qu et al., 2017; Jiang, 2021). Particularly, the COVID-19 pandemic has exacerbated AB among college students due to the restrictive lockdown measures (Hu, 2022; Toubasi et al., 2022; Ge, 2023). Much research indicated that the levels of AB among college students significantly increased during the COVID-19 pandemic (e.g., Jiang, 2021; Hu, 2022; Svavarsdottir et al., 2023). Moreover, with mental anxiety caused by the COVID-19 pandemic, the trend of “lying down” has become increasingly prevalent among Chinese college students such as not thinking ahead, decadent abandonment, and being weak daily (Jiang, 2021; Deng, 2022; Xu and Dai, 2022).

In order to enhance students’ educational ability, the Chinese government has strategically proposed building world-class universities and disciplines, referred to as the double first-class construction. According to this construction, Chinese universities are categorized into “Double First-Class” and “Double Non” (Non “Double First-Class”), where “Double First-Class” involves the top-ranked universities whereas “Double Non” refers to general universities or colleges (Chen, 2022; Lu and Wen, 2023). It is noted that there exists an evident gap between “Double First-Class” universities and “Double Non” universities or colleges within Chinese educational system (Chen, 2022). Compared with “Double First-Class” college students, “Double Non” college students are more likely to face AB due to lower school satisfaction and after-degree employment ratio (Zhao et al., 2019; Chen, 2023). Therefore, it is necessary to reduce the AB of “Double Non” college students, whereby enhancing the overall academic performance among Chinese college students.

Physical exercise (PE) refers to frequently physical activity with a certain intensity and duration (Zhao et al., 2022). Active participation in PE is not only beneficial to the physical and mental health of individuals, improving cognitive and non-cognitive factors (Zhou and Liu, 2022; Li, 2023), but also reduces the level of AB (Zhou, 2011). However, to some extent, the mechanism of PE influencing AB remains unclear. Domestic and foreign scholars (e.g., Castelli et al., 2014; Fu et al., 2023) have conducted much research on this issue, indicating that PE can negatively predict AB through self-efficacy and psychological capital, and the higher the amount of exercise is, the more obvious the predictive effect is. Yet, AB is a complicated psychological state, and the mechanism regarding how PE influences AB has not been fully verified.

Additionally, mobile phone addiction (MPA) is a common behavior among college students (Matar Boumosleh and Jaalouk, 2017; Sunday et al., 2021; Diotaiuti et al., 2022a,b). An empirical analysis based on the survey of 2,240 colleges and universities in China revealed that nearly 70% of college students are over-dependent on mobile phones (Rong, 2018). MPA not only affects the physical and mental health of college students, but also has a great impact on learning engagement (LE) and AB (Schaufeli et al., 2002a; Matar Boumosleh and Jaalouk, 2017; Sunday et al., 2021). However, research demonstrated that physical activity and mobile phone addition are interrelated with each other and physical activity could be considered as an intervention in lessening mobile phone addition (e.g., Azam et al., 2020; Li et al., 2021; Chen et al., 2022). In view of such relations among these variables, the present study aims to examine the structural relationships between PE, MPA, LE, and AB among Chinese “Double Non” college students.

## Literature review

### Academic burnout

Academic burnout is viewed as a persistent, negative, and passive mental state toward learning, which refers to students’ emotional and physical exhaustion, depersonalization, reduced enthusiasm for learning, or other psychological factors during their educational experience (e.g., Wang, 2008; Qu et al., 2017; Li et al., 2021). It has been a global problem affecting students’ mental health and academic achievement as it restricts students from psychological adjustment (Wang, 2008; Qu et al., 2017; Diotaiuti et al., 2021). Students with higher levels of AB are inclined to drop out or experience lower wellbeing and poorer mental health (Liu et al., 2020). AB may result from activities concerned with study, such as attending classes, submitting assignments, working with deadlines, and learning for long hours (Cazan, 2015).

Given that AB exerts a harmful effect on student development, researchers explored antecedents that may provide intervention for it and found that some factors may contribute to AB, while others may help reduce it (e.g., Salanova et al., 2010; Sunday et al., 2021). AB may be accelerated by MPA, which brings about physical and mental problems to students, leading to AB (Qu et al., 2017). However, LE is generally taken as a way to prevent AB (Jeon et al., 2022). Also, Tating et al. (2023) systematically synthesized studies of interventions for AB and concluded the following factors in research: Qigong exercises, progressive muscle relaxation, autogenic and laughter therapy, didactic behavioral sessions focusing on personal and professional development, and coping skills enhancement. However, the effects of these preventions on AB are short-term and unclear. In fact, AB results from both personal characters and external environment, thus comprehensive measures should be adopted to lessen or prevent AB.

### Learning engagement

Learning engagement is an important factor predicting students’ academic achievement, reducing their boredom and disaffection, and improving the quality of education (Schaufeli et al., 2002a). It is generally classified into multidimensional constructs, including behavioral engagement (e.g., students’ classroom behavior, time on-task, and concentration), emotional engagement (e.g., enjoyment of school), and cognitive engagement (e.g., investment in learning), according to Owen et al. (2018). But researchers do not come to a consensus on some themes of LE. Fredricks et al. (2004) suggested that cognitive engagement means the investment in learning, which is divided into subthemes of motivation, strategic learning skills, and problem solving. Further, Johar et al. (2023) systematically analyzed studies regarding student engagement and suggested that research should incorporate multifaceted types of LE such as social, cognitive, collaborative, behavioral, and emotional engagement. Therefore, it is considered that LE is a multidimensional concept.

Learning engagement has been an increasing hot topic as it is one of the most influential factors in promoting academic performance (e.g., Owen et al., 2018; Gao et al., 2021). Abbott-Chapman et al. (2013) investigated the relations among LE, adult education and occupation outcomes, and found that LE has long-term benefits for students who are more actively engaged in learning, as they are more likely to get better academic performance and tend to achieve better career and economic success in future life. Particularly, LE and AB are highly interrelated variables. Cazan (2015) demonstrated that there are significant and negative correlations between AB and LE among undergraduate students. Salanova et al. (2010) investigated obstacles and facilitators predicting academic performance among 527 college students, and found that students who feel more motivated and engaged in learning activities are more likely to take a positive attitude toward their study, leading to lower levels of AB.

### Mobile phone addiction

Mobile phone addiction is usually defined as excessive use of phones that causes adverse consequences, or disordered capabilities to control emotions or behaviors (Leung, 2008; Li et al., 2021; Sunday et al., 2021). The definition takes into consideration of the overuse of mobile phones without control. As mobile phones get more and more popular and become an integral part of the society, MPA turns out to be a heated topic for researchers (e.g., Yen et al., 2008; Fu et al., 2023). MPA is categorized as a type of behavioral addiction that means people cannot go a moment without a phone, and adverse consequences result from excessive use of phones, or they may experience symptoms similar to withdrawal when using the phone (Yen et al., 2008; Takao et al., 2009).

It has been found that anyone can be at risk of phone addiction, but research showed that behavioral problems and disorders resulting from improperly using mobile phone are commonly reported among young people (Gutiérrez et al., 2016; Sahu et al., 2019). Sahu et al. (2019) described characteristics of phone addiction that young people addicted to mobile phones share a fear of being without a mobile phone, disconnected or off the internet. It is an anxiety of receiving and responding immediately to text messages, or the false sensation of having received a text message or call, which leads to constantly checking the device. Meanwhile, Gutiérrez et al. (2016) revealed that patterns of mobile phone use differ between young boys and girls. Girls typically use their phones mainly for social interactions, while boys use phones for this purpose as well as accessing gaming applications. It has also showed that males tend to use their phones in risky situations and are at a higher risk of developing phone addiction (Roberts et al., 2014). Phone addiction is also related to age. Some studies found that the total time spent on mobile phones decreases with age, with the highest times reported for young people because of their lower self-control levels (Bianchi and Phillips, 2005; Yen et al., 2008).

Excessive use of mobile phones with difficulty to control affects basic activities of daily life, leading to negative consequences (Park and Lee, 2012; Lin et al., 2021; Fu et al., 2023). The more addicted to mobile phones college students are, the more negative impacts they experience (Lin et al., 2021), such as less LE (e.g., Matar Boumosleh and Jaalouk, 2017; Sunday et al., 2021) and more AB (e.g., Zhang et al., 2021; Fu et al., 2023). Gao et al. (2021) also empirically demonstrated that college students with higher levels of MPA tend to possess lower levels of LE. In terms of time, excessive use of mobile phones takes large amount of time that should have been spent on studying and disrupts daily schedule for study (Qu et al., 2017). Besides, MPA reduces sleep quality, consuming individuals’ learning energy and attention, and thus decreasing their LE (Matar Boumosleh and Jaalouk, 2017). Azam et al. (2020) carried out a systematic procedure of search and selected 8 cross-sectional studies to find the consistent support for positive outcomes of PE on MPA among adolescent and young students. That means MPA can be reduced by increasing participation and involvement in PE among the student population.

Researchers also examined the complex relationship between MPA and AB in details (e.g., Wood et al., 2012; Qu et al., 2017). It was found that excessive use of mobile phones takes up a lot of time for learning and rest, affecting normal daily routines and increasing fatigue (Wood et al., 2012; Rosen et al., 2013). Research has shown that MPA is negatively correlated with sleep and self-control, which are then closely related to learning outcomes. Bedsides, research shows that there is a direct relationship between phone addiction and AB, and MPA exerts a negative effect on AB (e.g., Li et al., 2021). Additionally, MPA may reduce LE in college students (Zhang et al., 2021). Generally, evidence confirmed that MPA decreases LE and leads to AB (e.g., Qu et al., 2017; Zhang et al., 2021).

### Physical exercise

Physical exercise refers to physical activities that are progressively, frequently, and consistently accomplished at a certain intensity (Pan et al., 2021). The non-disposable type of the activity could be planned and developed to not only maintain physical fitness and mental health, such as improving cardiopulmonary function and muscle strength and flexibility (Puetz et al., 2006), but also enhance academic performance (Wunsch et al., 2021). Benefits from minimal physical activity are obviously observed among sedentary people who are physically inactive and use mobile phones excessively (Xie et al., 2019).

It was found that the relationship between PE and the use of cellphones is complicated. Some studies showed that there is no relationship between the amount of daily PE and phone addiction in samples of college students or other adults (Barkley and Lepp, 2016; Fennell et al., 2019). While Chen et al. (2022) investigated the impact of PE on MPA and found that physical activity is an important external factor negatively affecting college students’ MPA. The relationship between PE and MPA is also affected by exercise intensity (Rebold et al., 2016). Generally, much more studies tend to indicate that physical activity is an important external factor in alleviating MPA (e.g., Li et al., 2021; Yang et al., 2021; Chen et al., 2022; Zhao et al., 2022).

In addition, a number of studies reported that PE is also associated with LE (Owen et al., 2016, 2018; Pan et al., 2021). Owen et al. (2018) empirically demonstrated that moderate-intensity activity has a positive relationship with LE while vigorous-intensity activity is less beneficial for LE in terms of activity intense. Whitt-Glover et al. (2011) also proved that students who regularly take part in physically activities are more engaged with their classroom lessons, indicating that PE has a positive influence on LE. However, Katz et al. (2010) empirically showed that physical activity has no effect on LE. Actually, there is a complicated association between physical activity and LE depending on the intensity of PE (Owen et al., 2016).

Physical activity brings about numerous benefits in education such as decreasing AB (e.g., Li et al., 2021; Chen et al., 2022). Fu et al. (2023) demonstrated that PE amount is directly and negatively associated with the AB of adolescents. However, even though boys have significantly higher levels of PE than girls, there is no significant gender differences in AB. Tong et al. (2021) also showed that PE plays an important role in reducing students’ AB by improving interpersonal relationships. In addition, previous research showed that PE has a significant effect on students’ mental health, which could in turn ease their AB (Gerber et al., 2013). Besides, the relations between PE and AB are affected by activity-intense. College students with higher level of PE show lower levels of AB than those with moderate or low intense of PE (Chen et al., 2022). Generally, PE may prevent or reduce AB directly as well as indirectly through the potential mediation effect (Fu et al., 2023).

Increasing evidence showed that PE is an interventional strategy in dealing with MPA, promoting LE and reducing AB (e.g., Castelli et al., 2014; Owen et al., 2018). However, LE may be interfered by MPA (e.g., Gao et al., 2021; Sunday et al., 2021). There are complicated relations among PE, MPA, LE, and AB. Although existing research has broadly involved these variables, there are still limited studies exploring the relationships between these four variables, simultaneously.

### The present study

Previous research has discussed the relationships between variables of PE, MPA, LE, and AB. However, few studies focused on the educational phenomenon that Chinese “Double Non” college students are more vulnerable to suffer from AB due to lower school satisfaction and after-degree employment ratio. The present study aimed to examine the structural relationships of PE, MPA, LE, and AB among Chinese “Double Non” college students, and explore corresponding mechanism to provide supportive guidance for alleviating potential AB.

Based on the existent literature, the following hypotheses were proposed.

H_1_: Physical exercise is negatively related to AB (a) and MPA (b), but positively related to LE (c).

H_2_: Mobile phone addiction has a positive effect on AB (a) but a negative effect on LE (b).

H_3_: Learning engagement is negatively associated with AB.

H_4_: The effect of PE on AB can be mediated by MPA (a) and LE (b), individually, and also by the serial mediating role of MPA and LE (c).

Gender, grade, and income were incorporated as covariates in the current model, which were theoretically and empirically relevant to AB according to previous studies (e.g., Luo et al., 2016; Du, 2020; Ye et al., 2021).

**FIGURE 1:**
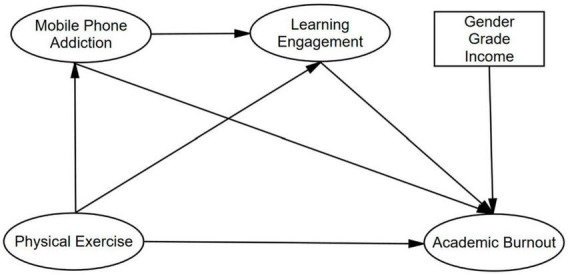
Hypothesized model.

The hypothesized model was shown as Figure 1.

## Materials and methods

### Participants

The participants were selected from two “Double Non” universities in Shandong Province, China. These universities are in the middle level among all “Double Non” colleges or universities in China, with respect to their size and academic quality. The cluster sampling method was used in the present study. In this study, participants of freshmen, sophomores, juniors, and seniors were all assigned the paper version of the questionnaire. From March to April 2023, when all participants were in the second half of the 2022–2023 academic year, we sent requests to the counselors of the prospective participants’ classes, inviting the counselors to assist us in recruiting participants from their classes. Freshmen, sophomores, and juniors consisted of 12, 8, and 9 classes of the participating universities, respectively, whereas seniors consisted of only 1 class of the participating universities, because they were generally doing internships off campus. All of the participants were enrolled voluntarily and informed about the purpose of study, the data confidentiality, and their right to withdraw from the study at any time. In each case, the questionnaires completed by the participants took approximately 10 min. A total of 1,019 undergraduate students agreed to participate in our study and 973 questionnaires were returned, with a response rate of 95.49%. After deleting missing data and outliers, the final sample contained 930 respondents for data analysis (272 men and 658 women; 378 freshmen, 234 sophomores, 286 juniors, and 32 seniors).

### Measures

#### Physical exercise

The Physical Exercise Rating Scale (PARS-3) compiled by Japanese scholar Hashimoto (1990) and revised by Liang (1994) of Wuhan Institute of Physical Education, was selected to measure PE in the current study. In this scale, the exercise intensity, exercise time, and exercise frequency were evaluated with a 5-point Likert scale. The scores of each index were recorded from 1 to 5 points, and higher scale scores corresponded to more PE. In this study, the Cronbach α coefficients of the PARS-3 scale was 0.70.

#### Mobile phone addiction

The Chinese version of the Mobile Phone Dependence Index Scale (MPDIS) compiled by Leung (2008) and revised by Huang et al. (2014) of the Chinese University of Hong Kong, was selected to measure MPA in the present study. It has 17 items with factorial components of the scale, including inability to control craving, feeling anxious and lost, withdrawal or escape, and productivity loss. A five-point Likert scale was used: 1 = “not at all,” 2 = “rarely,” 3 = “occasionally,” 4 = “often,” 5 = “always.” In this study, the Cronbach α coefficients of the MPDIS scale was 0.92, and the Cronbach α coefficients of each dimension was 0.81, 0.84, 0.77, and 0.84, respectively.

#### Learning engagement

The Utrecht Work Engagement Scale-Student (UWESS) developed by Schaufeli et al. (2002b) and revised by Fang et al. (2008) was selected to measure LE in the current study. It has 17 items with factorial components of the scale, including vigor, dedication, and absorption. A seven-point Likert scale was used ranging from 0 (never) to 6 (always, every day). In this study, the Cronbach α coefficients of the UWESS scale was 0.97, and the Cronbach α coefficients of each dimension was 0.90, 0.94, and 0.92, respectively.

#### Academic burnout

The Maslach Burnout Inventory-Student Survey (MBISS) compiled by Schaufeli et al. (2002b) and revised by Wu et al. (2010) was selected to measure AB in the present study. It has 16 items with factorial components of the scale: including emotional exhaustion, cynicism, and academic efficacy. Responses were provided on a five-point Likert scale with higher values referencing more frequent occurrences. In this study, the Cronbach α coefficients of the MBISS scale was 0.86, and the Cronbach α coefficients of each dimension was 0.73, 0.82, and 0.83, respectively.

### Analytical procedures

Models with latent variables were employed in the current study to explore the structural relationships among PE, MPA, LE, and AB, which may have the potential to increase measurement quality in contrast to models with semplice manifest variables (Rosel and Plewis, 2008). SPSS 25.0 was primarily used to export descriptive statistics and bivariate correlations. Mplus 8.3 software was used to estimate structural equation model (SEM) to examine various direct and indirect paths among latent variables. The bootstrapping bias-corrected confidence interval (CI) procedure was applied to the mediation analysis with 5,000 bootstrap samples (Hayes, 2009). The model fit was evaluated with absolute fit indices including Chi-squared test, root mean square error of approximation (RMSEA), and standardized root mean square residual (SRMR), as well as incremental fit indices such as comparative fit index (CFI) and Tucker-Lewi’s index (TLI). Given that the Chi-square statistic is sensitive to sample size, determined model fit standards are comprised of values more than 0.90 for CFI and TLI, as well as values less than 0.08 for RMSEA and SRMR (McDonald and Ho, 2002; Kline, 2005; Hooper et al., 2008).

## Results

### Descriptive statistics

Table 1 showed the results of descriptive statistics for all study variables. For PE, it displayed that male participants have a higher mean than females; freshman participants have the highest average whereas junior ones had the lowest; and those in the level 1 of income (more than 200,000 yuan) share the highest mean whereas those in the level 4 of income (less than 50,000 yuan) have the lowest. For MPA, it revealed that female participants have a higher mean than males; sophomore participants share the highest average while junior ones have the lowest; and those in the level 4 of income (less than 50,000 yuan) share the highest mean whereas those in the level 1 of income (more than 200,000 yuan) have the lowest. For LE, it demonstrated that female participants have a higher mean than males; junior participants have the highest average whereas sophomore ones have the lowest; and those in the level 2 of income (100,000–200,000 yuan) share the highest mean whereas those in the level 4 of income (less than 50,000 yuan) have the lowest. For AB, it indicated that male participants have a higher mean than females; sophomore participants share the highest average while junior ones have the lowest; and those in the level 4 of income (less than 50,000 yuan) share the highest mean whereas those in the level 1 of income (more than 200,000 yuan) have the lowest.

**TABLE 1:** Descriptive statistics and corresponding differences across demographic characteristics for latent variables.

Variables	PE	MPA	LE	AB
Total	2.63 (0.82)	2.84 (0.63)	4.81 (0.92)	2.66 (0.19)
**Gender**				
Male	3.14 (0.92)	2.79 (0.51)	4.73 (0.83)	2.68 (0.24)
Female	2.42 (0.78)	2.86 (0.49)	4.85 (0.87)	2.66 (0.21)
**Grade**				
Freshman	2.81 (0.89)	2.87 (0.49)	4.77 (0.94)	2.71 (0.22)
Sophomore	2.67 (0.91)	2.88 (0.55)	4.66 (0.98)	2.75 (0.22)
Junior	2.41 (0.72)	2.75 (0.49)	4.98 (0.95)	2.54 (0.26)
Senior	2.51 (0.87)	2.85 (0.48)	4.82 (0.77)	2.58 (0.25)
**Income**				
1	2.80 (0.80)	2.75 (0.61)	4.75 (0.97)	2.57 (0.27)
2	2.75 (0.82)	2.80 (0.47)	4.87 (0.91)	2.59 (0.23)
3	2.63 (0.79)	2.80 (0.51)	4.85 (0.93)	2.65 (0.24)
4	2.54 (0.84)	2.91 (0.51)	4.73 (0.96)	2.74 (0.69)

PE, physical exercise; MPA, mobile phone addiction; LE, learning engagement; AB, academic burnout.

### Assessment for the measurement model

The fit indices of the measurement model indicated that χ^2^ = 407.039, df = 86, RMSEA = 0.063, CFI = 0.937, TLI = 0.914, and SRMR = 0.047, revealing that the measurement model fit the data adequately (McDonald and Ho, 2002; Kline, 2005; Hooper et al., 2008). Table 2 showed that the bivariate correlations are significant among PE, MPA, LE, and AB, and the correlation coefficients range from −0.842 to 0.547, indicating that subsequent analyses are appropriate (Kline, 2005). Regarding the covariates, it was found that gender is significantly and negatively correlated with PE; grade is significantly and negatively linked to PE and AB, but significantly and positively related to LE; and income is significantly and negatively associated with PE, but significantly and positively correlated with MPA and AB.

**TABLE 2:** Bivariate correlations among all variables.

Variables	1	2	3	4	5	6	7
1. PE	_						
2. MPA	-0.133**	_					
3. LE	0.112**	-0.315***	_				
4. AB	-0.177***	0.547***	-0.842***	_			
5. Gender	-0.379***	0.042	0.054	-0.043	_		
6. Grade	-0.176***	-0.060	0.077*	-0.157***	0.056	_	
7. Income	-0.106**	0.075*	-0.041	0.137**	-0.007	0.052	_

*p < 0.05,

**p < 0.01,

***p < 0.001.

### Assessment for the structural model

**FIGURE 2:**
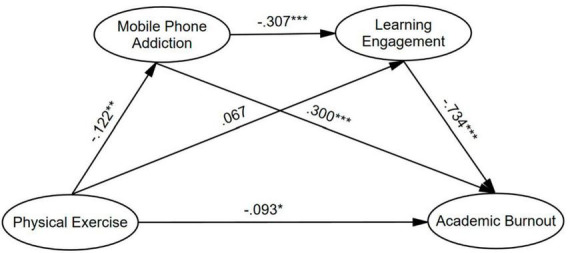
Structural equation model (*p < 0.05, **p < 0.01, ***p < 0.001).

The structural model displayed a satisfied fitness, indicating that χ^2^ = 604.534, df = 95, RMSEA = 0.076, CFI = 0.919, TLI = 0.900, SRMR = 0.067, according to previous studies (McDonald and Ho, 2002; Kline, 2005; Hooper et al., 2008). The results of the structural model assessment were demonstrated in Figure 2 and Table 3. The direct effects demonstrated that PE negatively and significantly predict AB (β = −0.093, *p* < 0.05) and MPA (β = −0.122, *p* < 0.01), but has no significant effect on LE (β = 0.067, *p* > 0.05). Further, MPA was a negatively significant predictor of LE (β = −0.307, *p* < 0.001) but a positively significant predictor for AB (β = 0.300, *p* < 0.001). Additionally, LE had a negatively significant effect on AB (β = −0.734, *p* < 0.001). With regard to the impacts of covariates on AB, it was found that AB is positively and significantly predicted by income (β = 0.086, *p* < 0.01) but negatively and significantly predicted by grade (β = −0.103, *p* < 0.01), whereas no significant association was reported between gender and AB (β = −0.038, *p* > 0.05).

**TABLE 3:** Summary of the model paths from the multiple mediation analysis.

Path/hypothesis	β	SE	CI (95%)	
			**Lower**	**Upper**
Gender→AB	-0.038	0.038	-0.113	0.035
Grade→AB	-0.103**	0.032	-0.170	-0.043
Income→AB	0.086**	0.031	0.026	0.146
**Direct effects**				
PE→AB (H_1*a*_)	-0.093*	0.040	-0.170	-0.016
PE→MPA (H_1*b*_)	-0.122**	0.042	-0.202	-0.039
PE→LE (H_1*c*_)	0.067	0.039	-0.010	0.142
MPA→AB (H_2*a*_)	0.300***	0.050	0.206	0.398
MPA→LE (H_2*b*_)	-0.307***	0.039	-0.378	-0.228
LE→AB (H_3_)	-0.734***	0.047	-0.812	-0.626
**Indirect effects**				
PE→MPA→AB (H_4*a*_)	-0.036*	0.015	-0.070	-0.012
PE→LE→AB (H_4*b*_)	-0.049	0.029	-0.107	0.007
PE→MPA→LE→AB (H_4*c*_)	-0.027**	0.010	-0.049	-0.009

Gender was coded as 0 = male, 1 = female; grade was coded as 1 = freshmen, 2 = sophomores, 3 = juniors, 4 = seniors; income was coded as 1 = more than 200,000 yuan, 2 = 100,000–200,000 yuan, 3 = 50,000–100,000, 4 = less than 50,000 yuan.

*p < 0.05,

**p < 0.01,

***p < 0.001.

### The mediation model analysis

The indirect effect is significant when zero is not between the lower and upper boundaries in the 95% CI, according to Hayes (2009). As seen in Figure 2 and Table 3, the effect of PE on AB was negatively and significantly mediated by MPA (β = −0.036, *p* < 0.05, 95% CI [−0.070, −0.012]). Further, the serial multiple mediation of both MPA and LE was statistically significant between PE and AB (β = −0.027, *p* < 0.01, 95% CI [−0.049, −0.009]). However, the path effect of PE on AB via LE was not significant (β = −0.049, *p* > 0.05, 95% CI [−0.107, 0.007]).

## Discussion

This study explored the structural relations of PE, MPA, LE, and AB among Chinese “Double Non” college students, adopting an SEM approach. The findings indicated that PE is significantly and negatively associated with AB and MPA, but not significantly related to LE; MPA has a significantly positive effect on AB but a significantly negative effect on LE; LE is significantly and negatively associated with AB. Also, the results showed that PE significantly and negatively influences AB through the mediation of MPA as well as the chain mediation of both MPA and LE. The findings were discussed below.

We tested the direct association between PE and AB, and found that PE exerts a significantly negative effect on AB of “Double Non” college students, which was consistent with those of previous studies (e.g., Gerber et al., 2013; Tong et al., 2021; Chen et al., 2022). Such a finding could probably be interpreted with two aspects. On the one hand, PE can produce a series of positive physiological changes for “Double Non” college students by relieving nervous tension, improving energy supply, and activating neurotransmitters of their bodies, which may reduce the perception of psychological pressure and alleviate physical and mental fatigue and depression, leading to the deceleration of AB (Gerber et al., 2013; Poirel, 2017). On the other hand, long-term systematic PE can not only improve the thinking ability and personal achievement, but also enhance the psychological experience by easing mental distress, emotional exhaustion and low sense of achievement for “Double Non” college students, thereby reducing their AB (Bretland and Thorsteinsson, 2015).

The results also demonstrated that MPA mediated the relationship between PE and AB, supporting the previous studies (Li et al., 2021; Yang et al., 2021; Chen et al., 2022). It was found that excessive use of mobile phones not only generates potential mental malaise and insufficient sleep among “Double Non” college students, but also damages their self-control ability and psychological capital, all of which tend to cause frustration in learning and then AB (Zhang et al., 2021). Whereas, according to the mindfulness re-perception model and the self-control resources model, “Double Non” college students who regularly participate in PE may have higher levels of mindfulness and self-control resources, which can promote the cognitive function and self-control ability, thereby reducing the dependence on mobile phones (Shapiro et al., 2006; He and Shi, 2015). Generally, PE alleviates AB by decreasing the behavior of MPA. In fact, “Double Non” college students are more likely to have relatively weak self-control ability and to indulge in mobile phone network, thus school administrators should take various measures to guide students to use mobile phones reasonably and cultivate their positive psychological quality.

The findings of the present study empirically revealed that the relationship between PE and AB is also mediated by the chain mediating role of MPA and LE. Although there was very little evidence for the chain mediating role of MPA and LE between PE and AB, research has confirmed the negative relationship between MPA and LE (e.g., Matar Boumosleh and Jaalouk, 2017; Qu et al., 2017; Lin et al., 2021). According to the theory of compensatory internet use, people tend to resort to network media such as mobile phones to relieve pain and discomfort when they experience negative emotions in life, which may lead to MPA (Kardefelt-Winther, 2014). Coupled with the randomness of excessive mobile phone use and potentially false pleasure brought by addiction to mobile phone use, “Double Non” college students may tend to escape from activities that require willpower and effort in reality, such as study and work. Consequently, it will reduce the LE, leading to a series of AB behaviors such as declining academic performance and skipping classes (Salanova et al., 2010; Cazan, 2015). MPA is considered as an important factor hindering LE among college students, thus LE may be enhanced by reducing MPA, with the help of PE, which may ultimately mitigate AB for Chinese “Double Non” college students.

The current findings indicated that although LE can significantly and negatively predict AB, the direct effect of PE on LE and the indirect effect of PE on AB mediated by LE are not significant, which was inconsistent of much previous research (e.g., Whitt-Glover et al., 2011; Castelli et al., 2014; Owen et al., 2018; Pan et al., 2021). Such an inconsistency may be caused by the distinctive learning background of Chinese “Double Non” colleges students. Although previous evidence indicated that PE significantly improves LE in adolescents (Castelli et al., 2014; Owen et al., 2018), PE does not necessarily promote LE in Chinese “Double Non” college students. Students in “Double Non” colleges generally have a middle or lower level of academic achievement compared with those in “Double First-Class” colleges. They also have a relatively lower level of motivation, initiative, and self-discipline in pursing academic performance (Zhang, 2020; Lu and Wen, 2023). In fact, majority of “Double Non” college students go after the principle of “pass long live,” that is, obtaining 60 points in a 100 points exam is their academic goal. It might be difficult for external factors to affect their LE. Moreover, according to self-determination theory (Ryan and Deci, 2017), external motivation, rather than internal motivation, has a more crucial effect on individuals’ psychological and behavioral outcomes. Therefore, the influence of external environment such as PE on LE may not be obvious. In addition, the non-significant effect of PE on LE among “Double Non” college students may also be related to the generally low exercise intensity in the survey. Therefore, further studies can be conducted on the relationship between PE and LE based on different participating groups to explore the potential boundary relationship between PE intensity and LE.

## Implications

This study may provide several practical implications for the impact of sport intervention on AB in Chinese “Double Non” college students. Firstly, “Double Non” college students generally have more free time to kill and experience more pressure due to less employment opportunities than “Double First-Class” college students in Chinese educational context, thus they are more likely to be addicted to mobile phones to relieve their psychological upset or even mental stress. In view of this, school administrators should increase opportunities for “Double Non” college students to participate in sports by optimizing physical education curriculum and adding burgeoning athletic projects such as yoga, which may enhance their interest and adaptability, transfer their psychological pressure, and then alleviate potential AB. Secondly, college managers should organize and implement programs to guide “Double Non” college students on how to rationally arrange their extracurricular life. In fact, these students are generally unaware of how to spend their college life to the fullest, leaving much spare time for themselves. Therefore, counselors may conduct the students to effectively utilize their spare time to strengthen PE, thereby decreasing AB. Thirdly, teachers in “Double Non” colleges may consciously guide students to learn independently, enhancing students’ self-control ability and restricting their unreasonable use of mobile phones in the classroom. Although the initiative of phone-free classroom is widely promoted in “Double Non” colleges, it is mainly implemented during the period of supervision and evaluation by the relevant administrative department. Therefore, a long-term and stable monitoring mechanism needs to be established to alleviate MPA among Chinese “Double Non” college students.

## Limitations and future directions

This study has some limitations. Firstly, PE, MPA, LE, and AB were all reported by students themselves in the present study. Therefore, future research may contain data from the perspectives of teachers, peers, and parents to decrease the potential social approval effect. Secondly, the current study adopted a quantitative analysis with the SEM approach. Further studies may contain qualitative information such as interview or observation data to comprehensively assess the role of PE for AB. Thirdly, the cross-sectional method in this study cannot directly infer the causal relationships among variables. Future directions may involve longitudinal research to examine the mechanism regarding how PE influences AB from a dynamic perspective.

## Conclusion

The current study examined the structural relationships between PE, MPA, LE, and AB among Chinese “Double Non” college students, with an SEM approach. The findings revealed that PE not only directly and negatively influences AB, but also indirectly and negatively influences AB through the mediation of MPA as well as through the chain mediation of both MPA and LE. Chinese “Double Non” college students should actively participate in PE during their educational experience, which is conducive to reducing MPA and then enhancing LE, whereby preventing or improving AB.

## Data availability statement

The raw data supporting the conclusions of this article will be made available by the authors, without undue reservation.

## Ethics statement

The studies involving humans were approved by the Qufu Normal University and Jining University. The studies were conducted in accordance with the local legislation and institutional requirements. The participants provided their written informed consent to participate in this study.

## Author contributions

CJ: Conceptualization, Data curation, Investigation, Software, Writing – original draft, Writing – review & editing. CF: Conceptualization, Formal analysis, Supervision, Validation, Writing – original draft, Writing – review & editing. JN: Conceptualization, Data curation, Methodology, Software, Writing – original draft, Writing – review & editing.
